# Intra-Uterine Pregnancy, Complicated with Extra-Uterine Fetation—Recovery

**Published:** 1878-05

**Authors:** 


INTRA-UTERINE PREGNANCY, COMPLICATED WITH EXTRA-UTERINE FETATION—RECOVERY.—Mrs. M., a large, rather tall, and an exceedingly robust woman, aged twenty-seven years, married four years, primipara, menstruated at sixteen, and enjoyed excellent and uninterrupted health; date of last menstruation sometime in November preceding my first visit; was seen by me March 26, 1876. I found her suffering most excruciating pain in the right iliac region. She had had pain in this locality during the past month at intervals, though not very severe and usually controlled by domestic remedies; she supposed herself to be pregnant. Inspection revealed a tumor rather low down in the right iliac region, as large as a fetal head, firm and solid, and encroaching somewhat upon the median line. The surface of the abdomen beyond the tumor presented nothing unusual.

A digital examination revealed the os soft and patulous, with but little development. There also existed right latero flexion to a moderate extent, with the uterus somewhat enlarged and adherent to the tumor. The uterine sound was not resorted to as a means of diagnosis. There were also present in the mammary glands changes indicating pregnancy, with morning sickness, etc.

On the 28th of March, two days after my first visit, there appeared a slight sanguineous discharge lasting for one day. The pain was controlled by opiates, and gradually disappeared after seven or eight days. The tumor continued to enlarge for about one month after my first visit, and then ceased to grow, and did not again take on development. At this time the tumor had attained the dimensions of a large fetal head. The case was seen and examined by several medical gentlemen, among whom contradictory opinions were entertained as to the nature of the tumor.

After a careful review of the history and symptoms above referred to, it appeared to me that I had one of two things to deal with, viz: either an ovarian tumor or extra-uterine pregnancy. The latter seemed to have the more evidence in its favor, and I adopted this diagnosis. After the cessation of the pain her condition was comparatively comfortable, but the uterus continued to enlarge after my first visit.

During the latter part of April, fetal movements were felt in the uterus, the outlines of which were well defined, and occupied a central position. This had no effect on the tumor other than to slightly obscure the outlines along its uterine border. Intra-uterine pregnancy was now a fixed fact, but the original tumor remained a matter of doubt. However, I was now inclined to change my diagnosis from extra-uterine pregnancy to intra-uterine pregnancy, complicated with ovarian tumor. I then anxiously awaited the period of the patient's accouchment.

On the morning of August 5th, I was summoned to attend the patient, and found her in the first stage of labor, the os dilated but little, and dilating slowly, with breech presentation, the uterine contractions being rather feeble. I now availed myself of the opportunity to inspect the abdomen during the contractions of the uterus, and the following is what occurred: Beginning with each contraction, there appeared a deep sulcus, or groove, along the line of contact of the uterine and iliac tumors, sufficient to receive a body as large as the index finger, the sulcus disappearing on the cessation of the contraction.

At four o'clock P.M. the dilatation was complete, membranes ruptured, and a moderate amount of liquor amnii discharged. The second stage of labor was protracted, owing partly to my inability to deliver the head promptly. At seven P.M. the patient was delivered of a dead female child, weighing about seven pounds, and well developed.

I had anticipated *post partum* hemorrhage. A short time before the second stage of labor was completed I administered ergot, and subsequently employed Crede's method for the separation and expulsion of the placenta; but in that I was disappointed. A rather free hemorrhage admonished me to remove the placenta immediately, which I did.

The uterine contractions during the third stage of labor were feeble, and on introducing my hand within the uterus, I discovered that the placenta was attached to the right lateral wall and fundus of the uterus, exactly at that point of the uterus in contact with the tumor. The placenta was with some difficulty detached and removed, after which considerable hemorrhage occurred from the want of sufficient uterine contractions. Hemorrhage continued to occur at intervals for the next two weeks, and was with difficulty controlled, though not alarming as to quantity at any time after the first twenty-four hours. Nevertheless, by its continuance, it rendered the patient's condition very critical, producing an extreme degree of anæmia and debility. The tumor remained much the same after the uterus was emptied, with the exception that it became more prominent and its outlines better defined.

Two weeks after labor, septicæmia set in, which defied all treatment, notwithstanding the most energetic measures were resorted to, both local and constitutional. After four weeks' treatment, with no improvement, and the patient's condition becoming daily more hopeless, with dissolution likely to occur at any hour, she became disgusted with treatment, disheartened with no prospects of recovery, she became reconciled to her fate, and refused to continue further treatment.

In this condition she remained some three weeks, gradually sinking lower and lower. The odor about her bed and person, which had hitherto been controlled during treatment by disinfectant solutions, now became so offensive that her friends could scarcely remain near her.

About the 15th of October, something was discovered protruding from the vulva. A physician was called in, one previously in consultation in the case, who removed a part of what proved to be the remains of a fetus, supposed to be near the fifth month of gestation, and in an advanced stage of decomposition; nevertheless, the placenta and cord were plainly discernible. It was also discovered that this product gained egress through the os uteri. Portions of this product continued to be discharged from time to time, together with pus and debris. The tumor undergoing marked diminution from the first escape of the putrid mass, and the general health and condition of the patient improving in a corresponding degree, the successful effort of nature to get rid of the offending mass, coupled with judicious treatment, enabled the patient to take a new lease on life, and four months from this time she was in the enjoyment of vigorous and robust health, with not a vestige of her former trouble remaining.

